# Non-fungible tokens (NFTs) in healthcare: a thematic analysis and research agenda

**DOI:** 10.3389/fdgth.2024.1377531

**Published:** 2024-06-11

**Authors:** Khulekani Sibanda, Patrick Ndayizigamiye, Hossana Twinomurinzi

**Affiliations:** ^1^ Department of Applied Information Systems, University of Johannesburg, Johannesburg, South Africa; ^2^ Centre for Applied Data Science, University of Johannesburg, Johannesburg, South Africa

**Keywords:** blockchain, healthcare, non-fungible tokens (NFTs), health data, patient privacy, research agenda, systematic review, data security

## Abstract

**Introduction:**

In the big data era, where corporations commodify health data, non-fungible tokens (NFTs) present a transformative avenue for patient empowerment and control. NFTs are unique digital assets on the blockchain, representing ownership of digital objects, including health data. By minting their data as NFTs, patients can track access, monetize its use, and build secure, private health information systems. However, research on NFTs in healthcare is in its infancy, warranting a comprehensive review.

**Methods:**

This study conducted a systematic literature review and thematic analysis of NFTs in healthcare to identify use cases, design models, and key challenges. Five multidisciplinary research databases (Scopus, Web of Science, Google Scholar, IEEE Explore, Elsevier Science Direct) were searched. The approach involved four stages: paper collection, inclusion/exclusion criteria application, screening, full-text reading, and quality assessment. A classification and coding framework was employed. Thematic analysis followed six steps: data familiarization, initial code generation, theme searching, theme review, theme definition/naming, and report production.

**Results:**

Analysis of 19 selected papers revealed three primary use cases: patient-centric data management, supply chain management for data provenance, and digital twin development. Notably, most solutions were prototypes or frameworks without real-world implementations. Four overarching themes emerged: data governance (ownership, tracking, privacy), data monetization (commercialization, incentivization, sharing), data protection, and data storage. The focus lies on user-controlled, private, and secure health data solutions. Additionally, data commodification is explored, with mechanisms proposed to incentivize data maintenance and sharing. NFTs are also suggested for tracking medical products in supply chains, ensuring data integrity and provenance. Ethereum and similar platforms dominate NFT minting, while compact NFT storage options are being explored for faster data access.

**Conclusion:**

NFTs offer significant potential for secure, traceable, decentralized healthcare data exchange systems. However, challenges exist, including dependence on blockchain, interoperability issues, and associated costs. The review identified research gaps, such as developing dual ownership models and data pricing strategies. Building an open standard for interoperability and adoption is crucial. The scalability, security, and privacy of NFT-backed healthcare applications require further investigation. Thus, this study proposes a research agenda for adopting NFTs in healthcare, focusing on governance, storage models, and perceptions.

## 1 Introduction

Non-fungible tokens (NFTs) are a particular type of token derived from Ethereum smart contracts (1, 2). NFTs differ from classical cryptocurrencies such as Bitcoin (3) because the latter are fungible and indistinguishable (4). In contrast, NFTs represent a unique entity (5). They represent digital or physical assets whose ownership is linked to an NFT. In recent years, NFTs have gained widespread attention in academia and industry, exploring their potential and business opportunities (2). NFTs are widely used in collectables, artwork, and gaming and are gaining broader traction in other areas (2, 6). Practical and functional NFT schemes rely on components such as blockchain and smart contracts (4). In the blockchain, all the participants, known as nodes, maintain a full copy of the blockchain. This property makes it more robust than a centralized system where, if the central node goes down, the whole system will be affected; it also removes the need for a trusted third party (3, 7). The distributed nature of blockchain and the elimination of a third-party appeals to researchers and implementers of blockchain. These properties create a robust system that provides a degree of anonymity and allows irreversible transactions to occur.

There has been an increased adoption of blockchain in non-financial areas (8), such as agriculture, supply chain management, and health and insurance. Blockchain provides a good framework for managing health data because of its robustness against attacks and failures and it also provides different access control methods (9). In addition, it can support data sharing and efficient audit trail management while allowing patients to control their records.

The most widely used blockchain platform for NFTs is Ethereum, which introduced the notion of smart contracts. Smart contracts were first introduced to facilitate trusted business activities without the use of third-party institutions (10). Smart contracts were developed in Ethereum to allow the dynamic addition of new features and business rules in domains outside of finance (11)⁠. A smart contract automatically permits the automation and execution of contractual obligations when certain conditions are satisfied by different parties. The smart contract is implemented on the blockchain to allow the recording of each statement as an immutable transaction⁠. The lifecycle of smart contracts consists of four phases. The first is creation, where parties negotiate and agree on the terms of the contract. The second is deployment, where the agreed-on contract is deployed on the blockchain (12). Since blockchain is immutable, the conditions of the contract cannot be changed. The third phase, the execution of the contract, depends on the conditions of the agreement, and the completion of the smart contract is the final phase. Most NFT implementations rely on smart contract-based blockchains for execution (4). With most assets and tokens relying on smart contracts, NFTs are also fully programmable.

The aim of this study was to to identify the challenges, gaps, and future research areas on using NFTs in healthcare. A thematic mapping of the landscape of NFTs in healthcare was also identified. To gain further insights into the development of NFTs in healthcare and to develop a research agenda, the following research questions were explored: What are the use cases of NFTs in healthcare? What are the current design models of NFTs in healthcare? What are the challenges and limitations of NFT-based applications in healthcare? What are the open research issues and areas for future research?

The study contributes to the literature by providing a comprehensive overview of the use cases, key challenges and limitations, and different design models of NFTs in healthcare. It also identifies four overarching themes: data governance (ownership, tracking, and privacy), data monetization (commercialization, incentivization, and sharing), data protection, and data storage. Lastly, it proposes a research agenda for adopting NFTs in healthcare, focusing on perceptions for commercializing medical data, storage models, and governance mechanisms for NFTs.

The remainder of the paper is structured as follows: section two provides a brief overview of related research;; section three outlines the research methods; section four describes the classification framework (how the research papers were selected and analyzed); section five contains the findings; section six presents the research themes; section seven discussion; section eight suggestions for future research; section nine presents the contributions and implications of the research; and finally, section ten comprises the conclusion and limitations of the study.

## 2 Related research

Several systematic reviews on applying NFTs in different fields have been published (13). NFTs have emerged as a transformative technology with implications for ownership, authenticity and digital asset management. A comprehensive review of the literature on NFT ownership reveals that blockchain technology cannot handle all copyright infringement challenges (14). Additionally, the literature shows that the most active research areas on the use of NFTs center on art and collectables, gaming, and decentralized finance (15). A bibliometric analysis and systematic review of NFTs and future research directions was conducted (16). Three distinct clusters of research activity, namely, blockchain technology, cryptocurrency, and digital art, were identified. Further, it was suggested that research into NFTs should be extended into other industries that have gained in interest and importance, such as healthcare (13, 16).

## 3 Methods

This study explored the state of the art in implementing NFTs in healthcare. A systematic literature review was conducted to identify published research on the topic. The approach followed four stages as outlined in (17) and the classification and coding framework by Amui et al. (18) was adopted.

### 3.1 Data sources and search strategy

The following electronic databases that host peer-reviewed multidisciplinary research articles were chosen as the sources:

• Google Scholar (http://scholar.google.com/).
• IEEE Explore (https://ieeexplore.ieee.org/Xplore/home.jsp).
• Elsevier Science Direct (https://www.sciencedirect.com).
• Scopus (https://www.scopus.com/).
• Web of Science (www.webofscience.com).

#### 3.1.1 First stage: collecting the related papers

The first step in the process was to search for relevant peer-reviewed articles based on the research questions. The search for relevant publications was performed using the search string defined below. The search string was developed from the research aim and the research questions. The search query was made as broad as possible to encompass a wide range of results related to the research of NFTs in healthcare. Six thousand two hundred and five (6,205) records were identified through search of which 6,003 came from Google Scholar. However, because of its limited search functionality and thus its tendency to return many non-relevant papers, only the first 100 relevant papers from Google Scholar were included in the study. The research string used was as follows:

(“Non-Fungible Tokens” **OR** “NFTs” **OR** “Digital Assets” **OR** “Blockchain Tokens”)

**AND**

(“Healthcare” **OR** “Medical Care” **OR** “Health Services” **OR** “Clinical Practice” **OR** “m-health” **OR** “mhealth” **OR** “ehealth” **OR** “e-health” **OR** “telehealth”)

The summary of the results returned for each database search is presented in Table 1.

**Table 1:** Summary of search results.

Database	Number of records	Number of records for full paper reading
IEEE	14	8
Scopus	22	13
Science Direct	146	2
Web of Science	20	7
Google Scholar	6,003 (100)	7

#### 3.1.2 Second stage: inclusion and exclusion criteria

The results from the online databases were filtered using inclusion and exclusion criteria. The inclusion criteria were as follows:

• Articles should be about the use of NFTs in healthcare.
• Articles should be either peer-reviewed journal articles or conference papers.
• Articles should be written in English.
• No specific time (publication timespan) was applied since NFTs are a new evolving technology.

The exclusion criteria were as follows:

• Articles focusing solely on NFTs but not discussing healthcare.
• Articles focusing solely on healthcare but not discussing NFTs.
• Articles without full-text availability.
• Editorials, news, discussion comments, and reviews.

#### 3.1.3 Third stage: practical screening

In this stage, non-duplicated articles from the second stage were excluded after reading the titles, abstracts, and keywords. The introduction and conclusion were read if the abstract was unclear. Articles that did not address the inclusion criteria were excluded. Two independent reviewers (KS and PN) read the studies' titles and abstracts. Discordant assessments were resolved through a discussion by the same authors or with the involvement of the third author (HT) when necessary.

#### 3.1.4 Fourth stage: full paper Reading and quality check

The last stage was full paper reading and quality check. The quality check was conducted using the following questions:

• Does the paper address the use of NFTs in healthcare?
• Are the objectives of the paper clearly defined?
• Does the paper clearly define the methodology used?

**Figure 1:**
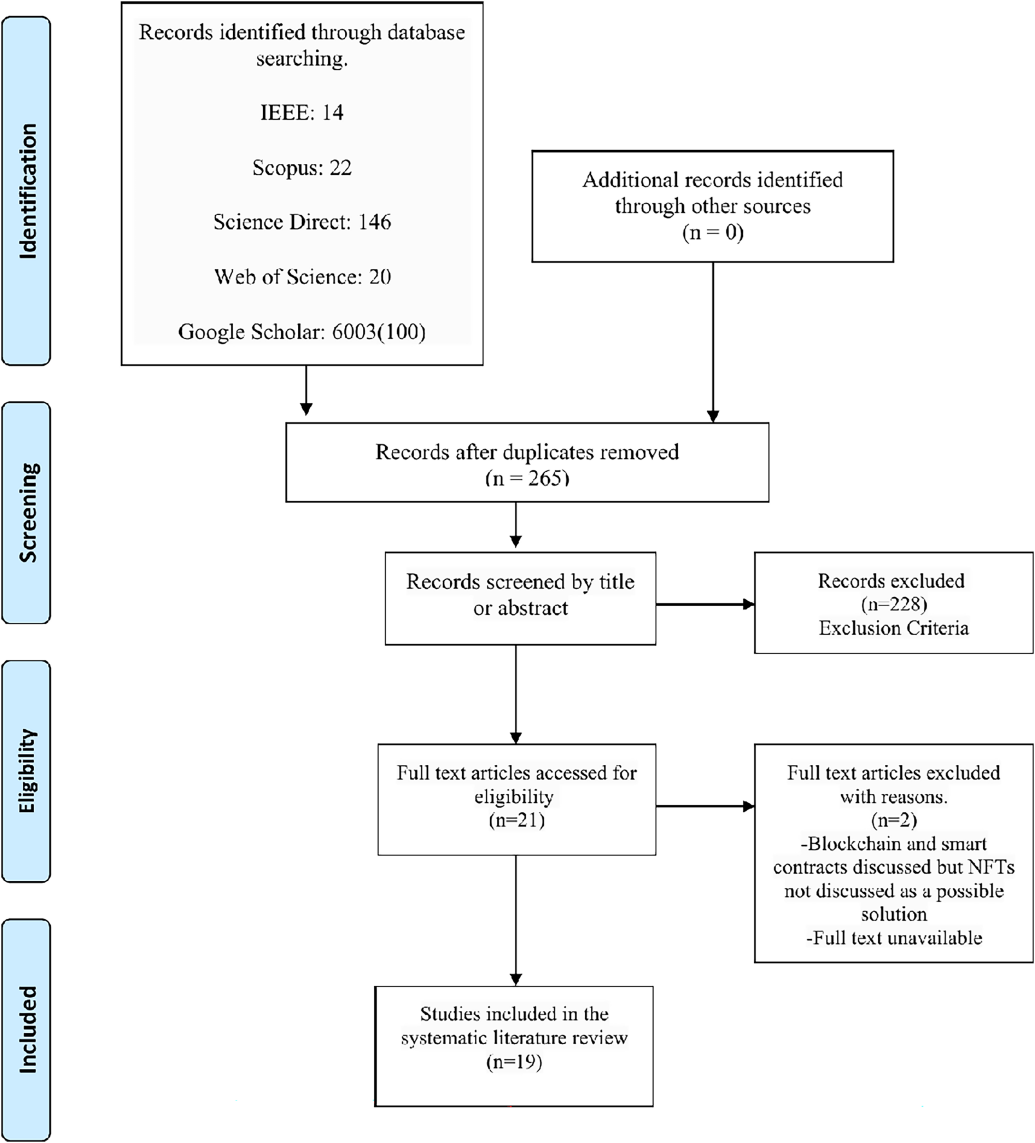
PRISMA flowchart of the search strategy.

Results from this stage show that 19 papers on the use of NFTs in healthcare meeting the stipulated criteria have been published. Compared to the extensive literature on Bitcoin, other cryptocurrencies, and blockchain use in healthcare, the research on NFTs in healthcare is in its infancy. An adapted PRISMA flowchart is shown in Figure 1.

## 4 Classification framework

The papers were classified according to context, method, paper type, scope, and origin. The list of classified papers is presented in Table 2. *Context* refers to the application setting in which the research was applied. In this study, categories were adopted as 1A (High-income country), 1B (Low-income country) and 1C (Not applicable) if the research could be applied in either setting. *Method* indicates the methodological approach of each research article: 2A (Qualitative research), 2B (Quantitative research) and 2C (Design research). *Paper type* indicates the contributions of the paper (19): 3A (Conceptual), 3B (Prototype) and 3C (Implementation). Conceptual papers propose new architectures, frameworks, or models. Prototype indicates that the concept has been further developed into a system prototype. Implementation indicates that the prototype has been integrated into a real-world healthcare system and is in use. *Scope* refers to the focus or the application of NFTs in healthcare. The scope was derived from reading the abstracts and extracting the keywords. A full paper reading was conducted to classify the papers into the identified categories. The identified categories were 4A (Patient-centric data management), 4B (Supply chain management) and 4C (Digital twin). *Origin* refers to the region in which the research was carried out. The categories were: 5A (America), 5B (Europe), 5C (Asia), 5D (Oceania) and 5E (Africa).

**Table 2:** Classification and coding of selected papers.

Authors	Method	Paper type	Scope	Origin
(Rai et al.) (20)	2C	3A	4A	5C
(Anjum et al.) (21)	2C	3B	4A	5C
(Ferone and Della Porta) (22)	2B	3A	4A	5B
(Zhuang et al.) (23)	2C	3A	4A	5C
(Shae and Tsai) (24)	2C	3B	4A	5C
(Gebreab et al.) (25)	2C	3A	4C	5C
(Gebreab et al.) (26)	2C	3A	4B, 4C	5C
(Bala et al.) (27)	2C	3B	4A	5C
(Musamih et al.) (28)	2C	3B	4B	5C
(Vijayalakshmi et al.) (29)	2C	3B	4A	5C
(Mohammed and Wahab) (30)	2B	3A	4A	5C
(Tanwar and Thakur) (31)	2B	3A	4A	5C
(Turki et al.) (32)	2C	3B	4B, 4C	5C
(Cunningham et al.) (33)	2C	3A	4A	5B
(Mahammadi et al.) (34)	2B	3A	4A	5C
(Chiacchio et al.) (35)	2C	3B	4B	5B
(Jayasinghe et al.) (36)	2C	3B	4A	5C
(Subramanian) (37)	2C	3B	4A	5A
(Sai et al.) (38)	2C	3B	4A	5C

## 5 Findings

The initial analysis of the selected papers is based on the classification framework outlined in section four. The focus is on the year of publication, publication channel, type, methodological approach, and scope of the study. The second phase of the analysis is a thematic analysis of the research papers.

### 5.1 Distribution of articles by year of publication, origin, and context

**Figure 2:**
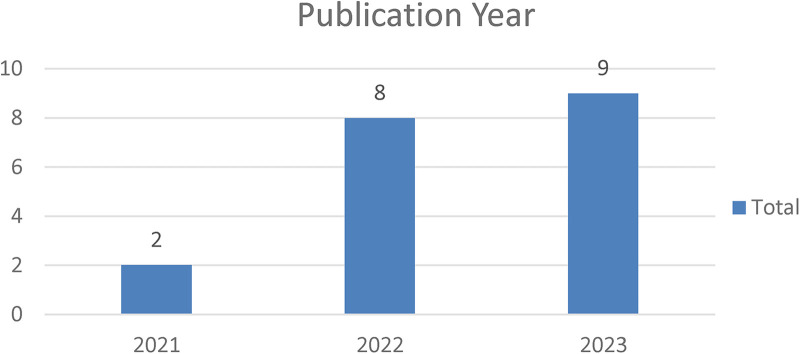
Articles by year of publication.

NFTs are a new technology with the potential to revolutionize healthcare. However, research on NFTs in healthcare is still in its early stages. Figure 2 shows the distribution of papers by year of publication. As indicated in the figure, there has been a significant increase in research in 2022 and 2023. In 2021, two papers focused on patient-centric data management were published (21, 24). There are still few publications on applications of NFTs in healthcare compared to publications focusing on implementing blockchain in healthcare—a core technology underlying NFTs (39). The countries of the host institutions of the researchers were used to show the geographic distribution of the research. Most of the research on NFTs has been conducted in Asia, with 15 of the 19 articles published in this area coming from Asian institutions.

All the NFT-backed blockchain solutions portrayed in the selected papers can be applied to suit any context, thus allowing for solutions developed in high-income contexts to be adopted in low-income ones. However, there is a lack of research on NFTs in healthcare in developing countries due to the emergent nature of this technology. These countries often prioritize addressing more pressing social and economic issues (17). Like blockchain, NFTs offer vast opportunities to solve complex social and economic problems, such as empowering and providing patient-centred healthcare (8, 19). NFTs can also be used to provide traceability in the drug supply chain to counter counterfeit drugs (40). Despite the early adoption of NFTs in other subdomains, such as art, collectables, and gaming, the healthcare sector still lags behind. More research on applying NFTs in healthcare needs to be conducted in developing countries to identify use cases.

### 5.2 Publication channel

The specific channels of publication are shown in Table 3. This information may help researchers identify journals and conferences to publish in or search for new research on NFTs in healthcare (19).

**Table 3:** Publication channel.

Publication channel	Authors
International Conference on Communication Systems and Network Technologies (CSNT)	(Rai et al.) (20)
Communications in Computer and Information Science	(Anjum et al.) (21)
Computer Communications	(Ferone and Della Porta) (22)
Computers in Biology and Medicine	(Zhuang et al.) (23)
IEEE International Conference on Cognitive Machine Intelligence (CogMI)	(Shae and Tsai) (24)
IEEE Access	(Gebreab et al.) (25, 26)
IEEE International Conference on Blockchain and Distributed Systems Security (ICBDS)	(Bala et al.) (27)
IEEE Transactions on Engineering Management	(Musamih et al.) (28)
International Conference on Trends in Electronics and Informatics (ICOEI)	(Vijayalakshmi et al.) (29)
International Journal of Online & Biomedical Engineering	(Mohammed and Wahab) (30)
Journal of Engineering and Applied Science	(Tanwar and Thakur) (31)
Journal of King Saud University—Computer and Information Sciences	(Turki et al.) (32)
Studies in Health Technology and Informatics	(Cunningham et al.) (33)
Technology Analysis and Strategic Management	(Mahammadi et al.) (34)
Applied Sciences (Switzerland)	(Chiacchio et al.) (35)
International Conference on Computing Communication and Networking Technologies (ICCCNT)	(Jayasinghe et al.) (36)
Journal of Medical Internet Research	(Subramanian) (37)
IEEE Internet of Things Journal	(Sai et al.) (38)

### 5.3 Paper type and methodological approach

**Figure 3:**
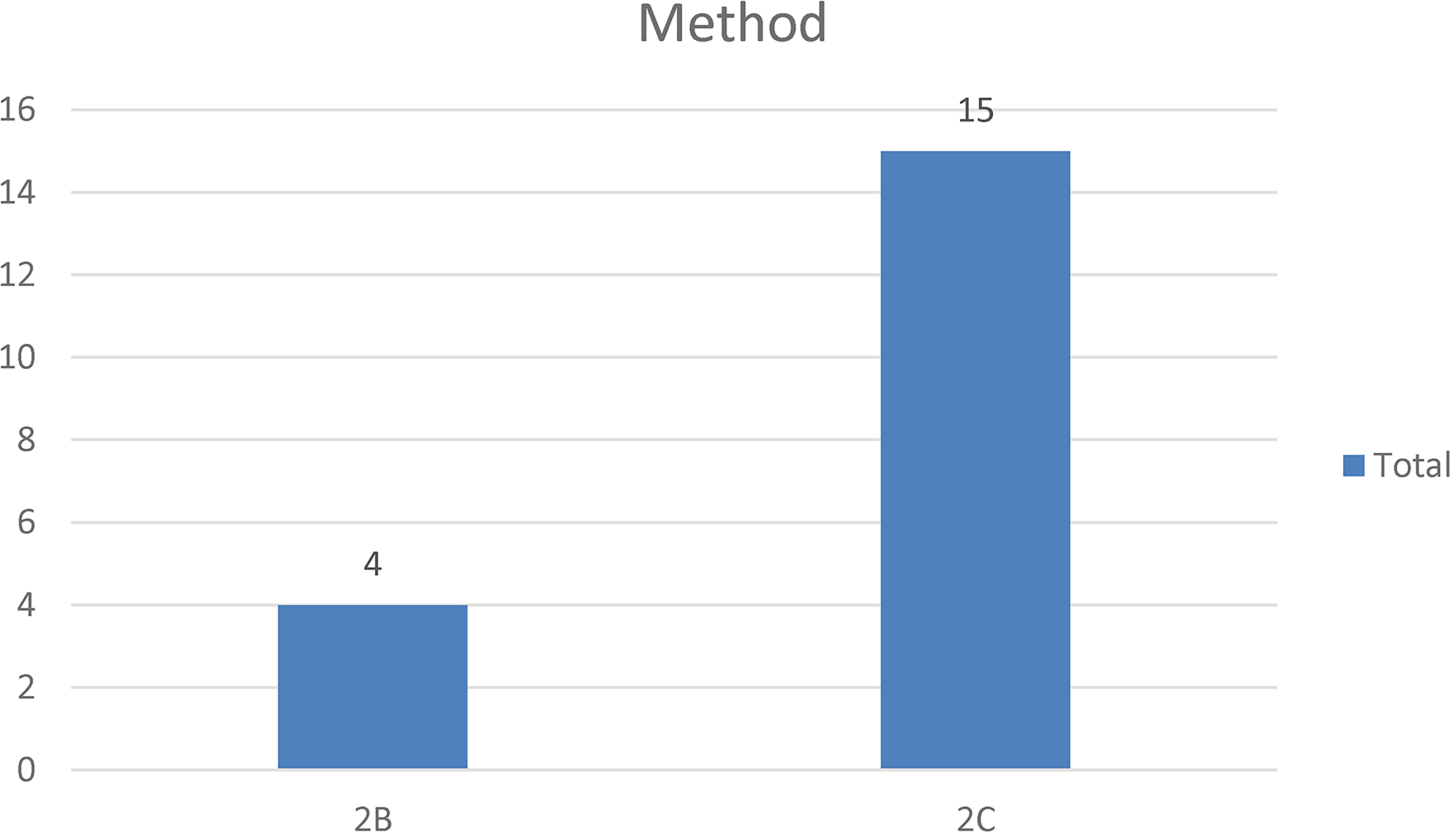
Methodological approach adopted in the selected studies.

This section reports on the types of papers selected and their methodological approach. In terms of the latter, and as shown in Figure 3, most (15) articles used a design science approach to designing and testing artefacts. The remaining four articles used a quantitative method with an experimental and descriptive focus. With NFTs being a new technology, designing qualitative research studies well-grounded in theory to generate meaningful insights is a challenge; hence, qualitative research was not used in the selected papers.

**Figure 4:**
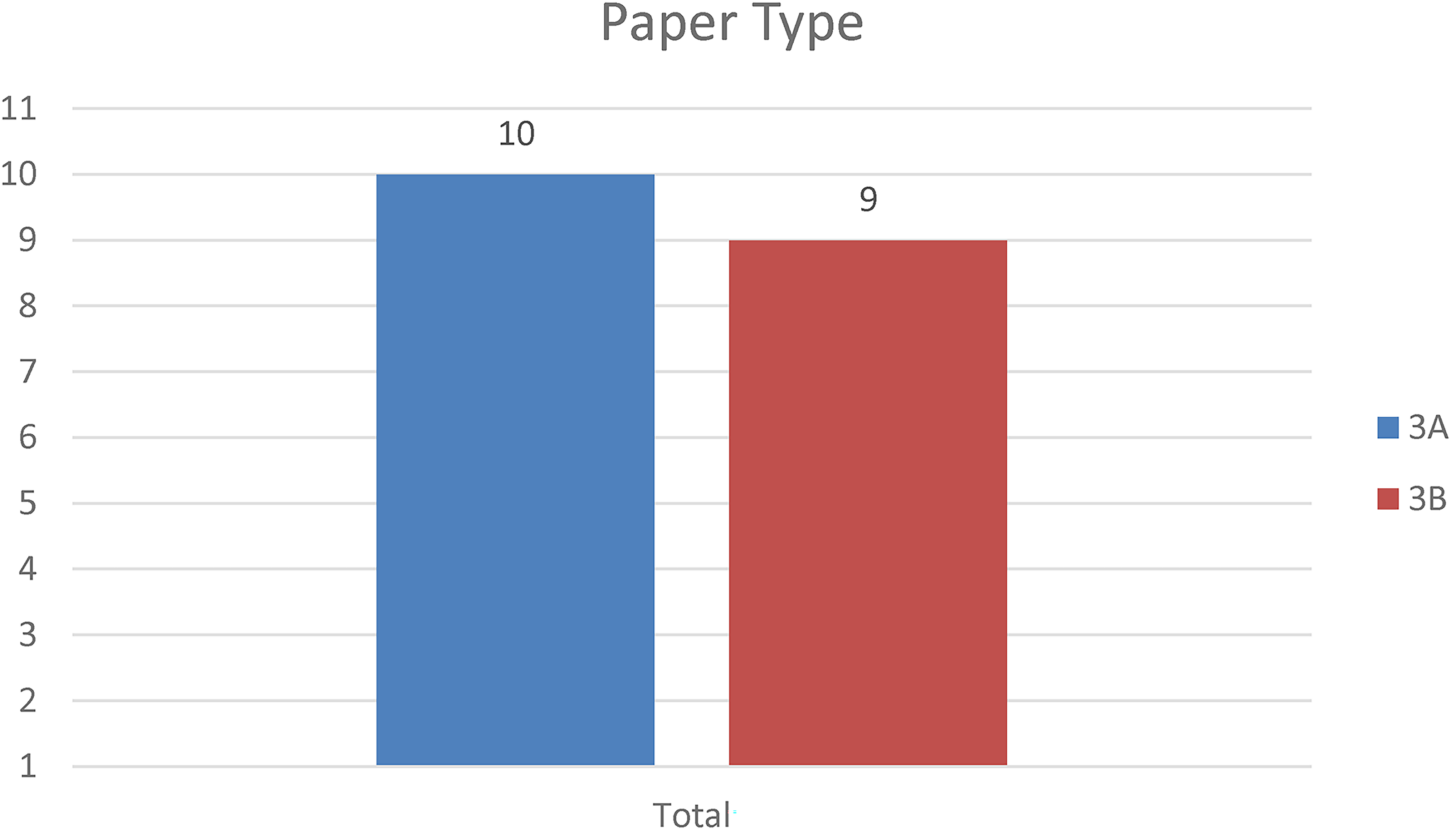
Paper type.

Figure 4 illustrates the distribution of papers by publication type. Ten papers developed concepts (models and frameworks), while nine concerned the development of fully-fledged prototypes. It is evident that the developed prototypes have yet to be implemented in real-world systems. The lack of implemented prototypes could be due to the development of NFTs in healthcare being in its infancy. The same challenges and obstacles faced in implementing blockchain technology (17) may also affect the implementation of NFTs. There is, therefore, a gap in the literature on identifying the challenges and obstacles preventing the implementation of NFTs in real-world healthcare systems.

### 5.4 Scope

**Figure 5:**
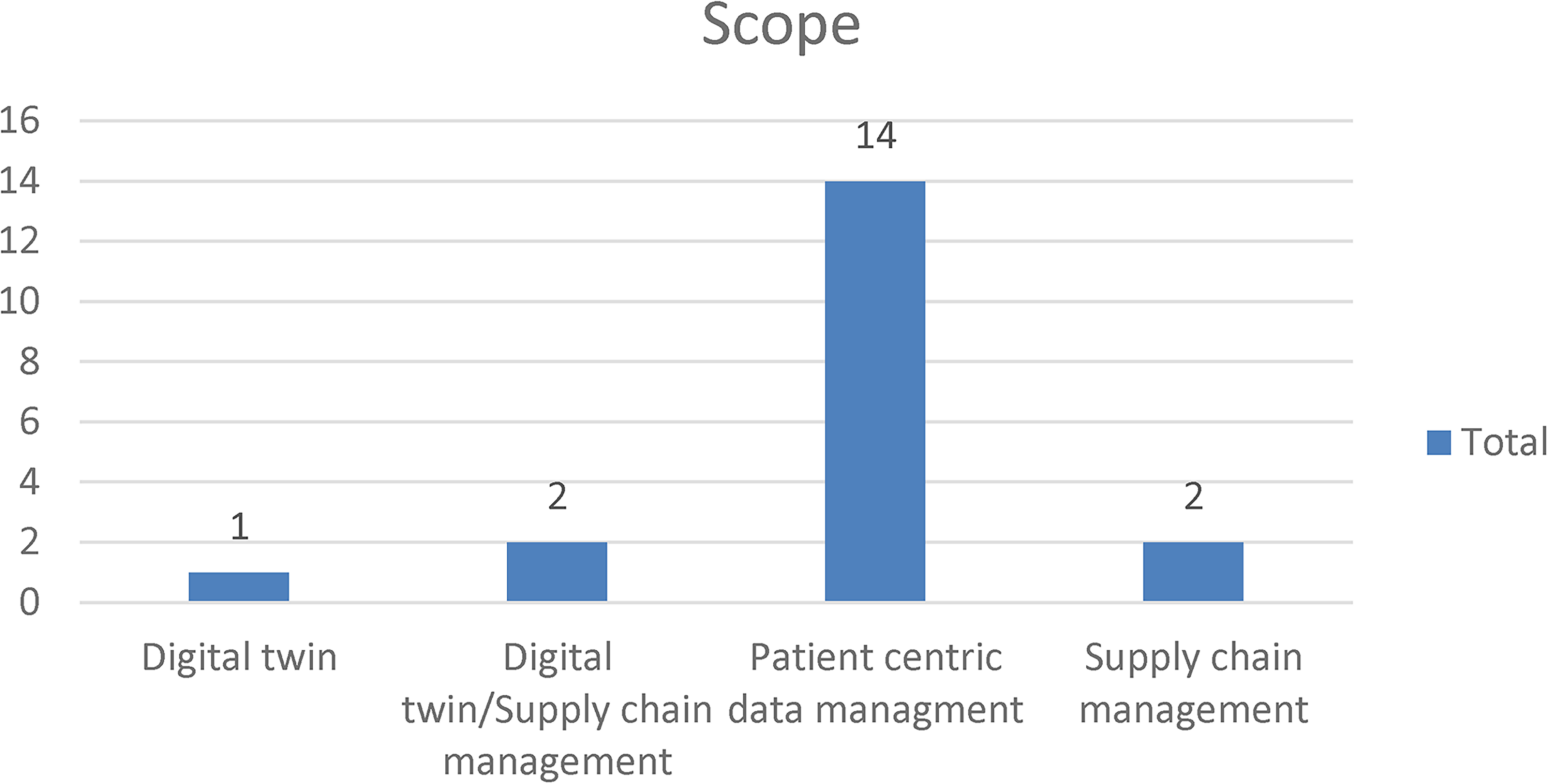
Application areas of NFTs in healthcare.

In terms of the scope of application of NFTs in healthcare, Figure 5 illustrates that 14 papers addressed the use of NFTs from a patient-centric data management perspective (19–23, 26, 28–30, 32, 33, 35–37). Four papers discussed using NFTs to track the provenance of healthcare products and services in a supply chain (26, 28, 32, 35). The aim of doing so was to ensure the authenticity and quality of the products through the supply chain. Three of the four papers represented the object being traced as a virtual product (digital twin) (25, 26, 32). The emergence of NFTs presents a unique opportunity to enhance transparency, traceability, security and streamlining of operations. However, further research and development are needed to develop clear and comprehensive regulations to facilitate the broader adoption of NFTs in healthcare. Furthermore, standardized data-sharing protocols and interoperability must be developed across different platforms.

## 6 Focal research themes

Thematic analysis was conducted following six steps: (1) familiarizing with the data, (2) generating initial codes, (3) searching for themes, (4) reviewing themes, (5) deﬁning and naming themes, and (6) producing the report (18).

In the first step, the papers were sorted by the application areas of NFTs. The papers were read several times to familiarize with the dataset before generating the codes. In the second step, codes were generated after analyzing, classifying, and comparing the data in the dataset. The third step comprised searching for themes—several codes identified in the preceding phase were integrated into a coherent theme guided by the research questions. In step four the identified themes were reviewed to determine whether they were distinct and supported in the entire dataset. In step five, the identified themes were defined and named to reflect the content of the data. In the final step the data were recorded for ease of analysis, and the main findings from each theme were summarized (producing the final report). The process yielded four themes related to NFTs in healthcare. The results are summarized in Table 4 and the mapping of articles to themes is presented in Table 5. In the section following Table 4, each theme is described.

**Table 4:** Integrated themes, underlying codes, and main findings.

Integrated themes	Underlying codes	Main findings
Data governance	• Data ownership • Traceability of data • Consent management	NFTs enable the ownership of data and allow the owners to transfer ownership to third parties while keeping track of who has access.
Data monetization	• Data commercialization • Data democratization	Health data can be minted as NFTs, thus allowing the data to be monetized and commercially traded. They allow easy and controlled sharing of data without the need for an intermediary.
Data protection	• Data security • Data privacy • Data immutability	NFTs are based on the blockchain platform; thus, they have inherited the same security, privacy, and immutability properties.
Data storage	• On-chain • Off-chain	Data can be stored on-chain if it is a small volume of data, such as metadata or hash values. Hash values would point to a large volume of data stored off-chain. On-chain data storage is very costly and leads to inefficient system operation.

**Table 5:** Mapping of papers to themes.

Authors	Data governance	Data monetization	Data protection	Data storage
(Rai et al.) (20)	✓		✓	✓
(Anjum et al.) (21)	✓		✓	✓
(Ferone and Della Porta) (22)	✓		✓	
(Zhuang et al.) (23)	✓		✓	
(Shae and Tsai) (24)	✓	✓	✓	
(Gebreab et al.) (25)	✓		✓	
(Gebreab et al.) (26)	✓		✓	
(Bala et al.) (27)	✓		✓	✓
(Musamih et al.) (28)	✓		✓	✓
(Vijayalakshmi et al.) (29)	✓		✓	✓
(Mohammed and Wahab) (30)	✓		✓	
(Tanwar and Thakur) (31)	✓		✓	
(Turki et al.) (32)	✓		✓	✓
(Cunningham et al.) (33)	✓			
(Mahammadi et al.) (34)	✓		✓	
(Chiacchio et al.) (35)	✓		✓	
(Jayasinghe et al.) (36)	✓		✓	✓
(Subramanian) (37)	✓	✓	✓	✓
(Sai et al.) (38)	✓	✓	✓	✓

### 6.1 Data governance

One of the most popular use cases of NFTs in healthcare is the management of medical records, such as electronic medical records (EMRs), which often contain sensitive information about patients undergoing treatment or post-treatment management. EMRs are frequently shared among stakeholders, from primary stakeholders such as health practitioners, pharmacists, and patients to secondary stakeholders, such as researchers and medical aid companies (41). The purpose of an EMR [also called an electronic health record (EHR)] system is to create, maintain, manage, and store client data while allowing access to only authorized users. Most of the papers (74%) in this systematic review focused on using NFTs to encapsulate EMRs to make patient-centric data management more effortless. This is consistent with other research (19) which focused on blockchain technology in healthcare. In this study, 48% of the 65 papers selected for review identified EMR use cases as the primary research topic in the literature. This consistency is not surprising since NFTs are built on blockchain technology. Due to their uniqueness, verifiability, and transferability, NFTs provide proof of data ownership.

Tokens are non-fungible and distinguishable, thus representing a tamper-proof and non-repudiable proof of data ownership (22)⁠. Patients can grant temporary ownership of their medical records to third parties and assign specific rights and access time to their NFTs (20). The unique nature of NFTs allows data traceability, whereby patients can view and track their records (23, 30, 34). The medicine supply chain stakeholders should provide a means to track and trace pharmaceutical supplies that pass along the supply chain (42). NFTs represent a perfect digital twin of any physical item that passes along a supply chain and provides for traceability processes (25, 26). An entity in the supply chain, the token owner, can invoke a smart contract through their private key to pass the token to the following entity in the supply chain. The next actor (entity) will be able to check the status of the NFT and verify the ownership of the previous entity. Counterfeit actors are identified if a deviation between the “real world” and what is on the digital ledger occurs (35)⁠. Lastly, regarding consent management, NFTs can represent patient consent. This can be done by recording and transmitting patient consent as NFTs (33). Using NFTs allows consent to access data to be verified for legitimacy without needing a third party. In this approach, patients can record their consent, allowing data consumers who may be researchers, insurance companies or other parties interested in health data to request data from data providers. Data providers are custodians of patient data, meaning they are responsible for storing and protecting it. Patients or data providers can revoke consent at any time.

### 6.2 Data democratization and monetization

Distributed ledger technology provides a data-sharing platform between providers and consumers (43). Data democratization refers to making data available to third parties that might require it. In this approach, NFTs enable patients or data providers to claim ownership of their records, which they can then share with other interested parties. Thus, NFTs empower patients to control their data and choose with whom they want to share the data. Access to patients' stored records can be through mobile applications (29). Data providers can view their medical information and track who can access it through NFTs. NFTs allow the dual ownership of patient data between the patient and the hospital, thus creating an avenue for the patient to derive value through monetizing their data. Several approaches have been proposed for using NFTs to incentivize healthcare data sharing. In one approach, data owners (patients and other stakeholders) can sell NFTs representing their healthcare data to third parties (37, 38). The price of the NFT can be determined based on the sensitivity of the data and the costs incurred in creating the NFT (44). In another approach, the patient is incentivized to maintain and share their records using several factors, such as data quality and relevance or willingness to share the data (24). The incentive can be tokens such as the ERC20 (37, 38). ERC20 tokens provide a platform for patients to be incentivized to share their data for research and development in a transparent, accountable, and secure way because the underlying blockchain technology offers transparency and accountability and fosters trust among stakeholders. However, it should be noted that blockchain, the underlying technology, suffers from scalability limitations, which may challenge large-scale data management in healthcare data.

### 6.3 Data protection

NFT-based health information systems can be implemented as an alternative to ensure data security, privacy, and immutability (36). Because NFT schemes are based on blockchain technology, they adopt the intrinsic properties of blockchain, such as immutability, which improves the security of health data since it cannot be easily corrupted, altered or accessed by a third party (32). All the health data on the blockchain is encrypted, time-stamped and appended in chronological order, and because the data is saved on a blockchain using cryptographic keys, it protects the patient's identity or privacy (27). Additionally, the blockchain platform's distributed nature improves NFT systems' availability. In the event of an attack, data can be gathered from other nodes where the data would have been replicated (19).

### 6.4 Data storage

Data can be stored on-chain if it is stored directly on the blockchain or off-chain if not. On-chain storage, however, leads to higher storage costs and scalability issues (28). Most NFT-based implementations store large volumes of data such as lab reports, x-rays, and other medical records off-chain on a decentralized platform such as the Interplanetary File System (IPFS), and referenced in the blockchain using a hash value to improve security and efficiency (21). In addition to enabling compact NFTs to be developed, storing data off-chain also enables quick access to data (20).

## 7 Discussion

This section explores the first three research questions in light of the study's results. The fourth research question is addressed in the section that follows, namely, suggestions for further research.

### 7.1 Rq1: what are the use cases of NFTs in healthcare?

The most common use case for NFTs in healthcare is patient-centric data management, with 73% of the published papers focusing on this topic. Other use cases include digital twins and supply chain management. However, the number of identified use cases is still relatively small compared to those for blockchain in healthcare. In a previous systematic review of blockchain in healthcare, five use cases were identified: data sharing, health records, access control, audit, and supply chain (9). Similarly, six different use cases, namely, EMR, pharmaceutical supply chain, biomedical research and education, remote patient monitoring, health insurance claims, and health data analytics, were identified in another systematic review (19). The fact that relatively few use cases for NFTs in healthcare have been identified indicates that this is still an emerging area of research and development. However, the potential benefits of NFTs in healthcare are significant, especially in biomedical research, where NFTs can be used to manage genomic data and provide integrity and transparency in clinical trials.

#### 7.1.1 Patient-centric data management

Patient-centric data management concerns data ownership, sharing, and monetizing health records (13). One of the most popular use cases of NFTs in healthcare is encapsulating EMRs as a token to facilitate patient-centric data management, with 14 of the 19 selected papers addressing this area. The properties of NFTs, such as data ownership, uniqueness, tokenization, monetization and verifiability, make them suitable for storing and managing patient records (13). Patient consent can be represented as an NFT that can be transferred to third parties as consent for data use (33). Similarly, architectures have been designed and developed that allow patients to share their records with third parties, with the latter providing a mechanism for incentivizing patients to share their health records (24, 30). To ensure integrity and non-repudiation, an extra layer of security in the form of QR codes can be added to secure the transfer of electronic records (36).

#### 7.1.2 Digital twin

A digital twin is a virtual representation of a physical object that takes inputs to produce outputs, which can be manipulated and analyzed to improve the physical object (13). Blockchain technology and NFTs can be utilized to create digital twins, providing a transparent, immutable platform and allowing for decentralized storage (13, 45). Medical devices can be digitally represented as NFTs with replacement parts and certificate documents embedded in a parent-child relationship (25). Moreover, these dynamic NFTs capture the reprocessing steps and can be used to authenticate and track the movement of refurbished medical devices. Furthermore, NFTs can represent medical devices as digital twins, capturing the devices' attributes and metadata throughout their lifecycle, from production to current use and ownership (26).

#### 7.1.3 Supply chain management

Supply chain management is the capability to monitor a product along its lifecycle from production, manufacturing, and distribution up to the stage in which it is delivered to the final user (35). Pharmaceutical supply chains face increased complexities due to the various entities in an increased networked environment and evolving digital world (40). Blockchain technology has been suggested as a possible solution to unravel the complexities in supply chains (40). NFTs can be used to develop a decentralized system to track and trace a product through its supply chain, thus improving the standard serializability process (35). The Internet of Things (IoT), blockchain and NFTs can enforce data provenance and integrity in the supply chain by tracking the movement of products and ensuring they are not tampered with (32).

### 7.2 Rq2: what are the current design models of NFTs in healthcare?

#### 7.2.1 Blockchain platforms

Thirteen of the 19 papers ⁠adopted the Ethereum platform to mint the NFTs. This finding is not surprising, given that Ethereum pioneered smart contracts in blockchain (4) and in terms of usability, Ethereum and Hyperledger are at the forefront (46) Smart contracts enable unfamiliar parties or decentralized participants to conduct exchanges without the need for a mediating third party. They allow communication between the user and the NFTs. Another component closely related to smart contracts is decentralized applications (DApps). DApps allow users to engage with smart contracts whilst abstracting on-chain data. One of the papers (23) used Quorum, a distributed ledger technology that is derived from Ethereum. Quorum uses a Raft-based consensus algorithm, which improves scalability and efficiency. Other platforms used are Hyperledger Burrow and Besu, which are used to verify the system's operation (22).

**Figure 6:**
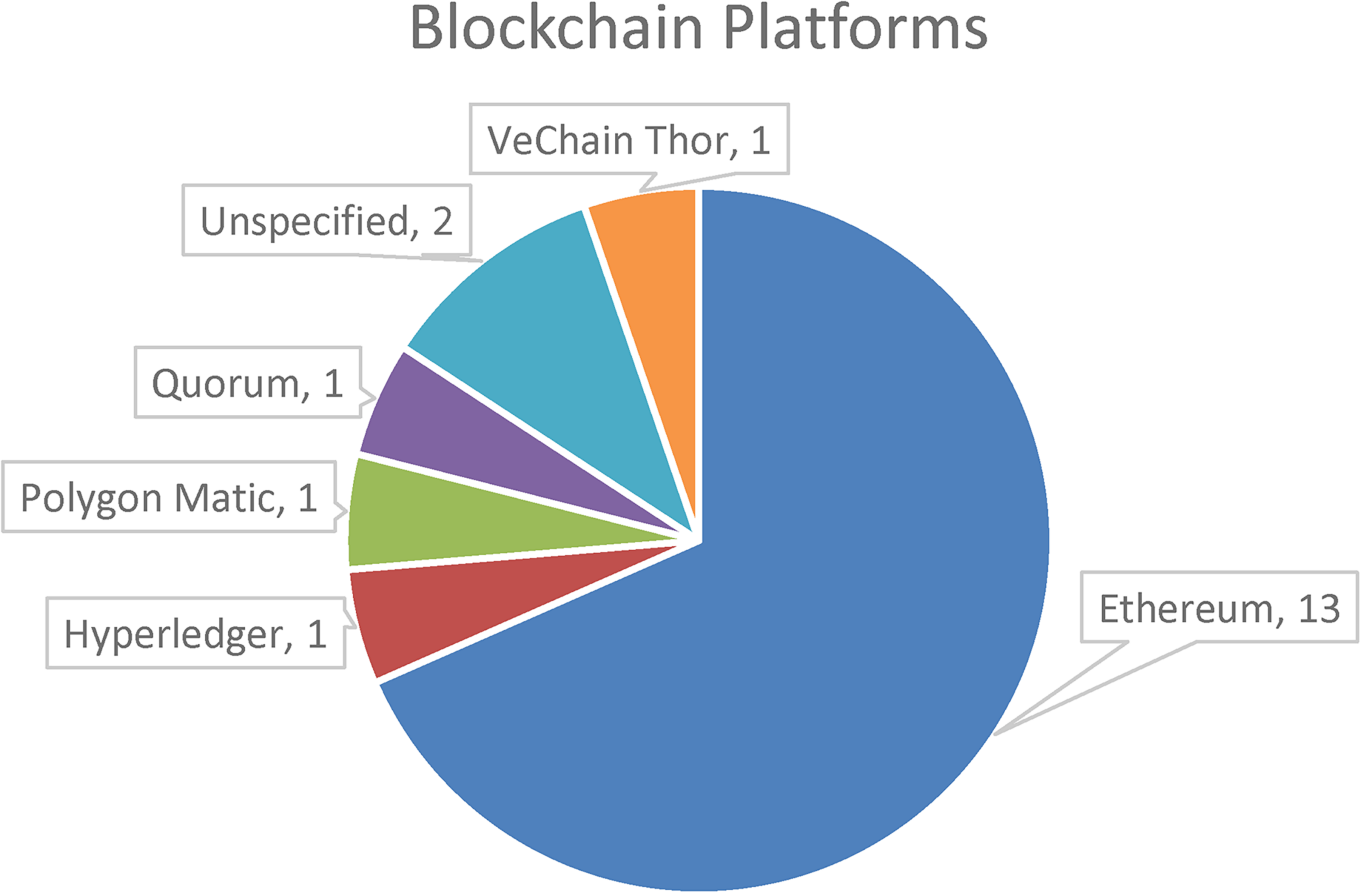
Blockchain platforms used in the selected papers.

It should be noted that since Hyperledger Fabric is an entirely permissioned network with robust security and privacy. It is primed to be the leading platform for developing healthcare applications where granular access control is critical (47). Figure 6 depicts the blockchain platforms used by the selected papers. Health informatics researchers and practitioners must evaluate the different blockchain platforms qualitatively and quantitatively before developing healthcare applications (48). Quantitative features such as scalability, cost, provenance, consensus, privacy, auditability, robustness, and qualitative features such as trust and governance should be considered (48).

It should be noted that there has been an increase in the number of other platforms minting NFTs. These platforms offer lower transaction costs and provide higher performance and examples are Solana, Flow, and Tezos (26). The increasing acceptance of alternative platforms provides a unique opportunity to advance healthcare NFT adoption. By leveraging on the reduced fees and quicker transactions offered by alternative platforms, the adoption of NFTs to transform data ownership, research, and patient engagement in healthcare can be accelerated. This transition to alternative platforms has significant implications for Ethereum, necessitating adaptation and innovation in this rapidly changing domain.

#### 7.2.2 Protocol standards

Two token standards are discussed in the selected papers: ERC-20 (49) and ERC-721 (50). The most common token standard is the ERC-20. It introduces fungible tokens that can be issued on top of Ethereum once the requirements prescribed by a smart contract are satisfied. The standard describes a set of rules adopted for a token deployed on the Ethereum ecosystem to function appropriately. Any ERC-20 token is indistinguishable from another token, differentiating it from an ERC-721 NFT that can represent unique entities. Earning the ERC-20 incentivizes data sharing between data owners, such as patients, and third parties who want to use patient records, uniquely represented as ERC-721 tokens (24).

#### 7.2.3 Storage

IPFS is a decentralized, content-addressable file storage system uniquely identifying each file with a hash value (21). This means that files can be stored without a central server. IPFS is often used with blockchain technology to ensure additional security and improve efficiency, where the file's hash value is stored on-chain, and the file itself is stored on IPFS (21). While centralized storage systems can be secure, they are more prone to problems such as single points of failure and loss of privacy (38). One application of IPFS is in the storage of NFT metadata (25). The researchers integrated IPFS with NFTs to store the metadata of each NFT to avoid the high cost of storing large files on-chain. However, it is essential to note that IPFS may have higher storage and retrieval costs (34) due to its need to replicate files across a network of nodes, which can require storage space and bandwidth.

### 7.3 Rq3: what are the challenges and limitations of NFT-based applications in healthcare?

The challenges NFTs face are due to them being coupled with blockchain platforms, leading to scalability issues (23, 37). As the number of transactions increases, latency becomes an issue, with few transactions processed per second due to network congestion (20, 25, 28). An increase in the number of transactions leads to a growth in Ethereum gas prices (28). NFT interoperability also poses a limitation to implementing data exchange between disparate systems. Most healthcare systems have different platforms that might need to interoperate (21).

Furthermore, the healthcare industry has heavily invested in infrastructure, and integrating blockchain and NFT-based systems with legacy systems poses challenges (28). A further limitation is the high cost of minting, trading, and pricing NFTs, which may impact their wide acceptance (25, 34). Poorly designed and implemented smart contracts pose a security risk in NFT-based systems. Malicious users can use bugs in smart contracts to execute unauthorized operations (32). Additionally, there is currently no standard way for coding a smart contract save for the ERC-721 standard for writing NFTs (28). Smart contract vulnerabilities and security risks thus remain the main challenges for NFT-based systems.

Using NFTs in healthcare also invokes many challenges in distributing and managing encryption keys (32). Key management places an overhead on the systems' operations and costs (21). The healthcare sector is highly regulated with centralized control to protect various stakeholders, especially patients. The issues of ethics and legality of granting and revoking patient consent outside a central authority are challenging ones (51). An additional challenge is engaging patients to share and manage their data. NFTs and blockchain are relatively new technologies; thus, they may lack experts (28), impacting their scalability.

## 8 Suggestions for further research

RQ4: What are the open research issues and areas for future research?

The research on NFTs has been concentrated on artwork, collectables, and assets, with applications in healthcare still in the early stages of development. Therefore, there is significant potential to explore open research questions and future directions for the use of NFTs in healthcare. This section will answer the fourth research question by proposing and setting research agendas.

### 8.1 Research agenda 1: the commercialization of medical data using NFTs

NFTs allow patients to take ownership and have more control over their data and how it can be utilized (38). Minting health data into NFTs will enable owners to track and decide with whom to share and sell their data. Organizations will also benefit from utilizing NFTs to incentivize patients to share their data for research. Other parties can also benefit from using health digital markets to access patient data directly (23). With all these identified benefits, further inquiry is needed to determine if patients would be willing to share their data with third parties, what data they would be willing to share, and the price and cost of minting.

On the other hand, organizations might not be willing to “share” the profits of ownership of health information. Therefore, there is a need to develop dual ownership models. Moreover, price determination of the data might also be a challenge. Hence, further research will be needed to develop pricing strategies for data owners and consumers. Another open issue that must be addressed is the cost of minting, trading, and pricing NFTs. With the development of the ERC-1,155 token standard (52), there is scope for decongesting the network and lowering the gas cost. For NFTs to be widely accepted in healthcare, there is a need for further development of platforms that offer lower costs for minting NFTs.

### 8.2 Research agenda 2: data storage models for NFTs in healthcare

Data is typically stored on-chain or off-chain. Storing large volumes of data on-chain is costly but more secure than off-chain storage (22). ⁠There is a need to research how to store data more efficiently either on-chain or off-chain with improved security. The challenges of scalability, security, and privacy of NFT-based applications are open issues which warrant further research.

### 8.3 Research agenda 3: governance mechanisms for NFTs in healthcare

Ownership of personal health data is a sensitive issue that needs further scrutiny. In the European Union (EU), legislation specifically regulates the ownership right of data. Thus, in the EU, secure access to data can be guaranteed but data cannot be owned (24). Like cryptocurrencies, NFTs also face the same barriers of strict management and the need for regulation from authorities (4). Further research is, therefore, needed on how to properly regulate the use of technology in healthcare while providing some form of autonomy when it comes to digital health data exchanges. The selected papers did provide various solutions, such as models, architectures, and prototypes, but these lacked implementation in practical settings. There is, thus, scope for further research to identify the challenges and obstacles limiting the implementation of prototypes in an actual medical setting.

Further, adoption frameworks for implementing the systems can also be explored. There is a need for an open standard to guarantee and speed up the interoperability of different NFT ecosystems. NFTs in healthcare are in their infancy, and the focus is on their feasibility. However, for NFTs to be fully adopted and deployed in practical healthcare environments, open standards for interoperability need to be defined. Ethical issues such as the right to be forgotten are all open research issues that need to be addressed to further the adoption of NFTs in healthcare.

## 9 Contributions and implications of the research

The following section explores the gap addressed in the study and its theoretical contributions and implications, and practical contributions.

### 9.1 Gap addressed in the study

Systematic reviews on the use of NFTs in healthcare are limited. Existing reviews (9, 19) have primarily focused on the blockchain, the underlying technology of the NFTs. Other research papers have explored the technical aspects and potential applications of NFTs in healthcare, but NFTs are still in the early stages of adoption in this field. In contrast, NFTs are widely used in other areas, such as art, collectables, and gaming (2). Therefore, a comprehensive systematic literature review on NFTs in healthcare was needed to identify use cases, design models, and challenges, and to set an agenda for future research.

### 9.2 Theoretical contributions

This study identified new areas of inquiry on the application of NFTs in healthcare and further explores new research questions. The study revealed that the primary use cases of NFTs in healthcare are patient-centric data management, supply chain management, and digital twin development. However, all the NFT-backed blockchain solutions identified in the review were developed in high-income countries, even though these solutions could be adapted to any context. There is a lack of research on NFTs in healthcare in developing countries, such as sub-Saharan Africa, which is missing out on opportunities to empower patients through NFT ownership of their medical records, improve supply chain provenance and integrity, and experiment with digital twins to improve healthcare systems.

Design science research methodology was used to design and test artefacts in most of the studies reviewed. However, many developed solutions were prototypes or conceptual frameworks without real-world implementation or evaluation. Therefore, to ensure more comprehensive acceptance of NFTs in healthcare, there is a need for real-world implementation and evaluation of such systems.

The Ethereum blockchain platform, along with some of its derivatives like Quorum, is the most widely used platform for minting NFTs, as Ethereum pioneered smart contracts. However, new platforms that offer lower transaction costs and higher performance, such as Solana, Flow, and Tezos, are now being explored.

Research on NFTs in healthcare is centred on four areas: data governance, monetization, protection, and storage. The focus is on developing solutions that empower patients to control their medical records while keeping them private and secure. Data monetization is also being explored to allow patient data to be minted as NFTs and made commercially available. Additionally, storage options are being explored to enable the development of compact NFTs that allow quick data access.

### 9.3 Theoretical implications

This study's findings could help to understand NFT adoption in healthcare. While three use cases of NFTs in healthcare were identified, more research is needed in other areas, such as genomic data management and clinical trial management. It is also worth noting that 79% of the papers studied used Ethereum to mint NFTs. Other platforms offering lower transaction costs and improved performance should be explored. As mentioned in Section 8 of this paper, other areas of investigation include stakeholder perceptions on the commercialization of healthcare data and governance considerations for NFTs in healthcare. Different data storage models appropriate for NFT implementation in healthcare could also be explored.

### 9.4 Practical implications

The findings of this study could be used to educate healthcare practitioners, decision-makers, patients, and all stakeholders on the potential benefits and challenges of using NFTs in healthcare. Additionally, the findings could be used to develop new products and services that use NFTs to improve health outcomes, such as secure, auditable, and patient-controlled access to healthcare information and improved drug supply chains.

There is a lack of research on adopting NFTs, data storage models and infrastructure, and governance issues in healthcare. Given the considerable advantages of NFTs in healthcare, it can be argued that all stakeholders, particularly policymakers, need to develop strategies to promote the adoption of NFTs in healthcare.

## 10 Conclusion and limitations

NFTs, as an emerging technology, offer a unique opportunity to enhance the healthcare system by providing a platform for exchanging healthcare information in a secure, traceable, and decentralized environment. This study demonstrates shows that NFTs have the potential to be used in various healthcare systems, to provide patient-centric services and to facilitate tasks such as the tracking and tracing of pharmaceutical products through the supply chain. However, for NFTs, as a new and developing technology to gain broader acceptance in practical settings, there is a need to develop standards and regulations to govern the exchange and management of healthcare data without a central authority.

It is vital to highlight the main limitations of the study. Firstly, only 19 articles from five scientific databases were selected for the systematic review, and this may raise concerns as grey publications such as technical reports and patents from the industry (which tend to publish their innovations through such channels) could have been missed. However, selecting papers from peer-reviewed channels helps to retrieve quality research articles. Secondly, NFTs are a new technology still to develop and warrants further research.

## Data Availability

The original contributions presented in the study are included in the article/supplementary material, further inquiries can be directed to the corresponding author.
